# SARS-CoV-2 Delta variant isolates from vaccinated individuals

**DOI:** 10.1186/s12864-022-08652-z

**Published:** 2022-06-04

**Authors:** Lauren Brinkac, Sheila Diepold, Shane Mitchell, Stephanie Sarnese, Lee F. Kolakowski, William M. Nelson, Katharine Jennings

**Affiliations:** 1 Noblis, Reston, VA 20191 USA; 2 Tetracore, Rockville, MD 20850 USA

**Keywords:** SARS-CoV-2, COVID-19, Delta B.1.617.2, Variant of concern, Mutation, Genome sequencing, Vaccine

## Abstract

**Background:**

The SARS-CoV-2 Delta variant was first identified in the U.S. in March 2021 and has rapidly become the predominant lineage across the U.S. due to increased transmissibility, immune evasion and vaccine breakthrough. The aim of this study was to better understand the genetic diversity and the potential impact of mutations observed in SARS-CoV-2 viruses circulating in the U.S. in vaccinated individuals.

**Results:**

Whole genome sequencing was performed on thirty-four SARS-CoV-2 positive samples using the Oxford Nanopore MinION. Evolutionary genomic analysis revealed two novel mutations, ORF1b:V2354F and a premature stop codon, ORF7a:Q94*, identified in a cluster of SARS-CoV-2 Delta isolates collected from vaccinated individuals in Colorado. The ORF1b:V2354F mutation, corresponding to NSP15:V303F, may induce a conformational change and result in a disruption to a flanking beta-sheet structure. The premature stop codon, ORF7a:Q94*, truncates the transmembrane protein and cytosolic tail used to mediate protein transport. This may affect protein localization to the ER-Golgi. In addition to these novel mutations, the cluster of vaccinated isolates contain an additional mutation in the spike protein, at position 112, compared to the Delta variant defining mutations. This mutation, S112L, exists in isolates previously obtained in the U.S. The S112L mutation substitutes a bulky hydrophobic side chain for a polar side chain, which results in a non-conservative substitution within the protein that may affect antibody-binding affinity. Additionally, the vaccinated cluster of isolates contains non-synonymous mutations within ORF8 and NSPs which further distinguish this cluster from the respective ancestral Delta variant.

**Conclusions:**

These results show there is an emerging sub-lineage of the ancestral Delta variant circulating in the U.S. As mutations emerge in constellations, those with a potentially beneficial advantage to the virus may continue to circulate while others will cease.

**Supplementary Information:**

The online version contains supplementary material available at 10.1186/s12864-022-08652-z.

## Background

Since the emergence of severe acute respiratory syndrome coronavirus 2 (SARS-CoV-2) at the end of 2019, variants carrying mutations across the genome have been appearing and spreading at an alarming rate. The Delta variant (PANGO lineage B.1.617.2 and sub-lineages AY.1, AY.2, and AY.3) has shown increased transmissibility [1, 2], reduced sensitivity to antibodies [3] and vaccine breakthrough in partially and fully vaccinated individuals [1, 2, 4, 5]*.* Breakthrough is expected because vaccines are not 100% effective, but the underlying causes of breakthrough afforded by genomic diversity within this lineage are not yet fully elucidated.

The evolution of Delta sub-lineages demonstrates the heightened fitness of these SARS-CoV-2 variants and their ability to persist in the population. Mutations are altering proteins and specific protein regions different from, or in addition to, those of previously prevalent variants such as Alpha B.1.1.7. A study to map the antibody-binding sites within the Spike (S) protein N-terminal domain (NTD), a known hotspot for mutations as well as glycosylation, described a “supersite” for neutralizing antibody binding [6]*.* The authors posit that selective pressure from host immune responses, specifically those that are antibody mediated, are responsible for frequent mutations and deletions in the NTD of circulating SARS-CoV-2 lineages. Two other SARS-CoV-2 proteins that stimulate antibody production are open reading frames ORF7a and ORF8 [7]. There is some evidence that both proteins interact with human immune mediators and aid immune evasion, which may drive the frequent mutations seen in these viral factors [8–10].

In this study, we identified a sub-lineage of the Delta variant circulating in the U.S., particularly in central states such as Colorado (CO), Texas (TX), and Wyoming (WY). This sub-lineage is defined by a spike protein mutation at position 112 that has been identified in lineages at low prevalence worldwide and in circulating U.S. isolates starting at end of April 2021. Within this sub-lineage we generated whole genome sequences of a cluster of SARS-CoV-2 isolates obtained from vaccinated individuals in CO between June 3 and June 21, 2021. This Delta S:S112L sub-lineage cluster co-harbors two novel mutations: ORF1b:V2354F, corresponding to nonstructural protein NSP15 at position 303 (NSP15:V303F), and a premature stop codon (Q94*) truncating ORF7a. In this study, we describe the functional and structural implications of the distinguishing mutations identified in these CO isolates. The core branch-defining mutations constituting this breakthrough cluster may have arisen from a single or multiple introduction event. Those mutations in constellations that have a potentially beneficial fitness advantage may persist in the circulating viral population and those with a reduced capacity to persist and will stop circulating soon after appearing.

## Results and discussion

Thirty-four positive SARS-CoV-2 isolates obtained from clinical nasal swab samples were collected in CO (*n* = 29), Virginia (VA) (*n* = 3) and Iowa (IA) (*n* = 2) between April 29, 2021 and June 21, 2021 and sequenced to completion (Additional file 1). Samples covered a range of viral titers; nucleocapsid (N) gene cycle threshold (Ct) values ranged from 19.3 to 35.3, envelope (E) gene Ct values ranged from 16.3 to 33.0 and RNA-dependent RNA polymerase (RdRp) Ct values ranged from 24.6 to 40.2 with RdRp as undetectable in one sample. Roughly equal numbers of males (*n* = 16) and females (*n* = 18) with birth years ranging from 1918 to 1992 were represented. The 28 samples were collected between June 3, 2021 and June 21, 2021 from vaccinated individuals in CO with the majority of the samples coming from a single facility. As all patient samples were collected by Clinical Laboratory Improvement Amendments (CLIA) laboratories, more-complete clinical data are not available due to the U.S. Health Insurance Portability and Accountability Act of 1996 (HIPAA) restrictions. This restriction limited the associations that could be made. Whole genome sequences covered > 99.6% of the Wuhan-Hu-1 reference genome with a mean coverage of 2300x, and reported mutations had a mean depth of reads spanning position (DPSP) of 2231x (Std Dev—3511) with a mean quality score of 57.8 (Std Dev – 34.86). All 34 genomes were represented by the B.1.617.2 lineage now dominant in the U.S.

### Delta sub-lineage defined by S protein NTD mutation

**Fig. 1:**
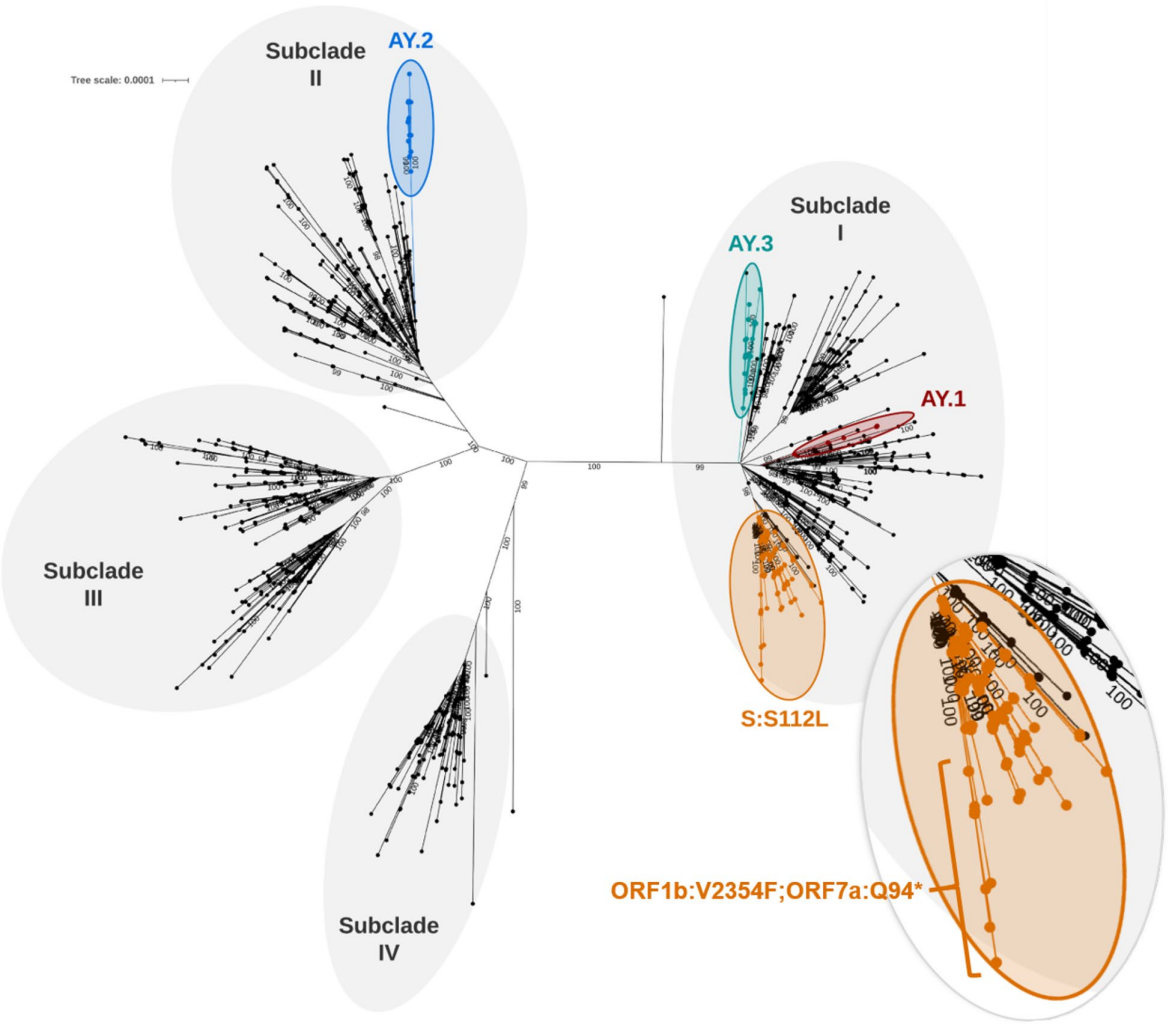
Phylogenetic analysis of U.S. SARS-CoV-2 Delta isolates. Unrooted maximum-likelihood phylogeny inferred from 1318 high quality unique SARS-CoV-2 genomes using Wuhan-Hu-1 (GenBank sequence MN908947.3) as the reference. Delta B.1.617.2 subclades and sub-lineages are highlighted and labeled. Branch defining CO cluster mutations labelled in a zoomed view of the S:S112L sub-lineage. Numbers at nodes represent > 98% bootstrap support. The scale bar represents the number of nucleotide substitutions per site

**Fig. 2:**
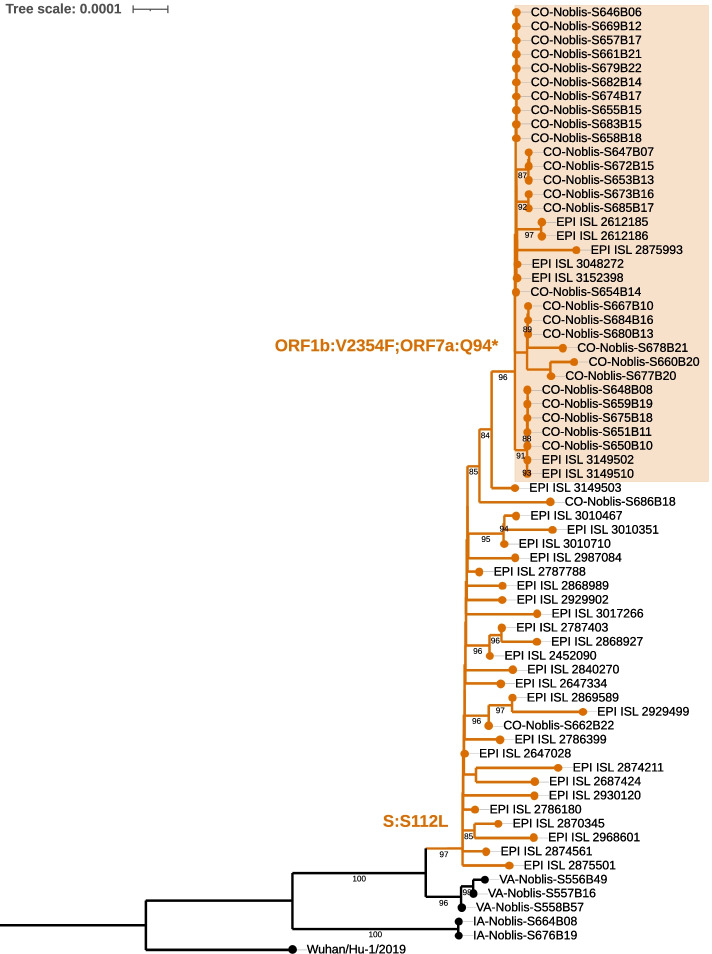
Phylogenetic analysis of U.S. SARS-CoV-2 Delta S:S112L sub-lineage isolates. Maximum-likelihood phylogeny inferred from 34 SARS-CoV-2 genomes analyzed in this study and 33 GISAID representative genomes belonging to the S:S112L sub-lineage using Wuhan-Hu-1 (GenBank sequence MN908947.3) as the reference. CO cluster isolates are highlighted, and branch-defining mutations labelled. Numbers at nodes represent > 80% bootstrap support. The scale bar represents the number of nucleotide substitutions per site

The Delta B.1.617.2 variant clusters into four distinct subclades (Fig. 1) [11]*.* Within those subclades, isolates that acquired an additional S:K417N have been designated AY.1 and AY.2, and a third AY.3 designation sharing canonical AY.1 mutations except S:417 and containing ORF1a mutations T3636A and I3731V [12]. All 29 CO and three VA isolates sequenced in this study cluster with Delta subclade I genomes while the two isolates collected in IA cluster with genome sequences belonging to Delta subclade II. We also identified sub-lineage defined by a serine to leucine substitution at position 112 in the NTD of the S protein (S:S112L) (Fig. 1) in 4.6% (*n* = 1,739) of the U.S. Delta variant genome sequences (*n* = 38,027) and part of subclade I. This sub-lineage consists of isolates collected as early as April 23, 2021, primarily from central U.S. states with 26% from CO (*n* = 455), 20% from TX (*n* = 353) and 8% from WY (*n* = 145). Clustered within the S:S112L sub-lineage, are a monophyletic group of 27 isolates obtained from vaccinated individuals in CO. These breakthrough cluster isolates co-harbor ORF1b:V2354F (NSP15:V303F) and ORF7a:Q94* mutations distinguishing them from other S:S112L Delta sub-lineage isolates (Fig. 2). This combination of mutations consisting of canonical Delta B.1.617.2, S:S112L, NSP15:V303F and ORF7a:Q94* mutations is the first reported occurrence of this constellation in the U.S. and worldwide. Included in this cluster are seven GISAID genomes obtained from individuals with unknown vaccination status in CO (*n* = 3), Kansas (KS) (*n* = 2), Florida (FL) (*n* = 1) and New Mexico (NM) (*n* = 1). Both the samples obtained from vaccinated individuals in this study and those obtained from GISAID were collected in June 2021 suggesting these variants were circulating primarily in the central U.S during the same timeframe. However, the exact phylogenetic placement of these GISAID isolates are unknown as their genomes contain stretches of low-quality sequence and a subset of their mutations could not be confirmed. Their inclusion in this phylogenetic analysis provides geographical and temporal context but should be interpreted with caution. Therefore, it is uncertain whether the mutations defining this cluster could have arisen from either a single or multiple introduction event. The closest phylogenetic neighbors to this cluster is an isolate obtained in CO (EPI_ISL_3149503) that contains the same core constellation except for NSP15:V303F, and one additional isolate obtained from a vaccinated individual and collected in the same CO location as those belonging to the breakthrough cluster but missing both NSP15:V303F and ORF7a:Q95* mutations. The additional NSP3 and NSP14 mutations identified in this isolate suggest it arose from a separate introduction event.

### Distinguishing non-synonymous mutations identified in Delta variants

**Fig. 3:**
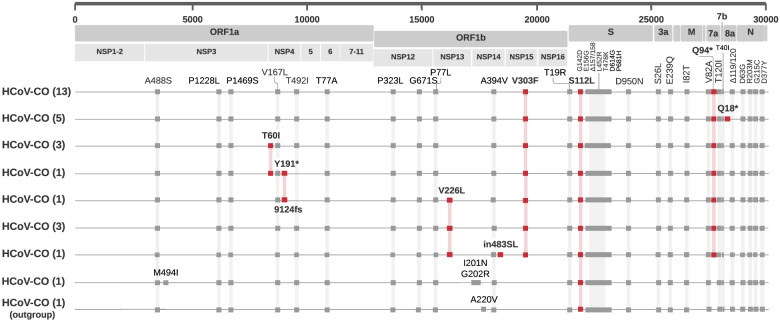
Distinct mutation constellations observed in CO isolates sequenced in this study. Mutations highlighted in red are the distinguishing mutations of the breakthrough cluster. The first eight constellations were identified in isolates obtained from vaccinated individuals from the same CO location. The ninth constellation labeled as outgroup, was obtained from a different location in CO from an individual with unknown vaccination status

We identified non-synonymous mutations in coding sequences of SARS-CoV-2 genomes from vaccinated individuals that distinguish them from canonical Delta B.1.617.2 variant mutations. These include mutations in the S protein, accessory proteins ORF7a and ORF8, and nonstructural proteins (NSPs) involved in RNA synthesis (Fig. 3). Eight distinct constellations of mutations occurring in these proteins were identified among the isolates obtained from vaccinated individuals, with 46% of the isolates harboring the same constellation. The next largest constellation was identified in 18%, followed by two additional constellations each represented by 10% of the SARS-CoV-2 genomes obtained and sequenced in this study.

### Spike protein

The S protein mediates attachment to human cell surface angiotensin converting enzyme 2 (ACE2) receptor for viral entry into host cells during infection [13, 14]. It consists of two subunits: S1 and S2. The S1 subunit contains an N-terminal domain (NTD) and receptor-binding domain (RBD) that bind directly to the ACE2 receptor, and the S2 subunit mediates virus-cell membrane fusion [14]. Isolates sequenced in this study contain the characteristic mutations identified in Delta variants circulating in the U.S. including, T19R, E156G, ΔE156-F157, L452R, T478K, D614G, P681R, and D950N [1], as well as the lesser prevalent G142D Delta mutation (Additional file 2) [6, 15]. The S112L mutation defines an emerging sub-lineage that might have evolved as a result of host antibody-mediated selective pressure given its location in the NTD [6, 15, 16]. The introduction of a bulky hydrophobic side chain may induce conformational changes to the strand configuration at this position as well as accessibility from a buried-to-exposed position affecting antibody-binding behavior. The role of this mutation in immune evasion has not yet been demonstrated.

### Accessory proteins

ORF7a is a member of the immunoglobulin (Ig) domain superfamily with studies suggesting an immune evasion role of SARS-CoV-2 ORF7a [10, 17]. The N-terminal signal peptide (amino acids [aa] 1–15) flanks the ORF7a luminal ectodomain (aa 16–96) which structurally possesses an Ig-like β-sandwich consisting of seven β-strands folded into two β-sheets, stabilized by cysteine disulfide bonds [10]. This ectodomain is highly conserved among the SARS-CoV ORF7a [17, 18]*.* At the C-terminal half, ORF7a consists of a transmembrane (TM) domain (aa 97–116) and a short cytoplasmic tail (aa 117–121) also called an endoplasmic reticulum (ER) retention motif, both of which are used to mediate protein transport to the ER-Golgi intermediate compartment [9, 17]*.* The SARS-CoV and SARS-CoV-2 cytoplasmic tail of ORF7a contains the sequence KRKTE [10]. Based on a prior study of SARS-CoV ORF7a, the triplet KRK is present in other proteins localized to the Golgi and appears to be required for recognition by the transport proteins for ER to Golgi translocation [18]*.* It appears that the Ig-like ectodomain (aa 1–96) binds with high affinity to CD14 + monocytes [10, 19]*. *In vitro studies with purified recombinant ORF7a ectodomain demonstrated that coincubation with CD14 + monocytes decreases HLA-DR/DP/DQ suggesting a reduced ability to present viral antigen to host immune cells [10]*.* In the same study, co-incubation of purified recombinant ORF7a ectodomain with CD14 + monocytes also upregulated expression of proinflammatory cytokines suggesting a role in the development of a cytokine dysregulation – a known risk factor for severe Coronavirus Disease 2019 (COVID-19) disease [20, 21]*.*

In Delta isolates, ORF7a mutations V82A and T120I are conserved. However, in the CO isolates discussed in this study, we also identified a premature stop codon at Q94 (Additional file 3). We hypothesize that truncation results in a secretory state for ORF7a as opposed to one that is normally Golgi-bound. The truncation presumably affects protein localization to the ER-Golgi, but possibly does not eliminate the functionality of the ectodomain, as suggested by CD14 + monocyte studies performed with purified recombinant ORF7a (aa 1–96) [10, 19]*.* Data from another member of the Ig-like domain family, ORF8, supports this suggestion because it has a similar genomic organization to ORF7a but naturally lacks a TM domain and cytoplasmic tail and is functional as a secretory protein [22]. The implication of a truncated state on immune modulation and localization are not yet known. Empirical, epidemiological, and clinical studies are needed to validate these inferences.

Last, the five Delta isolates collected in VA and IA were included for comparison. All three VA isolates contain an ORF7a mutation that is significantly truncated (L56*) due to a frameshift affecting residue 57. This mutation disrupts the four-stranded β-sheet of the β-sandwich, presumably causing a loss of function. In both IA isolates, the cytoplasmic tail is mutated to KRK**I**.

ORF8, like ORF7a, has an N-terminal signal sequence followed by an Ig-like domain [8, 17]*,* making it a member of the Ig domain superfamily*.* Unlike ORF7a, ORF8 has an approximately 35 aa insert between β-sheets three and four of the Ig-like domain, which also contains an added cysteine residue, and it lacks a TM domain and cytoplasmic tail [8, 17]. ORF8 is highly variable, which may be a viral strategy to counter immune pressure from the host [17, 19]. The crystal structure of ORF8 reveals an intermolecular disulfide bond between the Cys20 residues of two ORF8 monomers [8]*.* The symmetrical dimer involves several important interactions including hydrophobic interactions between V117 on each monomer, salt bridges between R115 and D119, and hydrogen bonding between F120 on one monomer and A51 and R52 of the opposite monomer [8]*.* Furthermore, ORF8 also forms a dimer through noncovalent, hydrophobic interactions between ORF8 monomers, which is unique to SARS-CoV-2 [8]*.* Conserved among the Delta variant isolates described in this study is the deletion at residues 119–120 in the ORF8 protein, except for five CO isolates that are differentiated by a premature stop-codon at position 18 of ORF8 (Additional file 4**)**. Given the importance of the residues between 117–120 on dimer stabilization, the deletion of residues 119–120 in the Delta lineage may indicate a reduced functional role for ORF8. Despite numerous and occasionally large deletions, such as a 382-nucleotide deletion in 45 of 191 diagnostic samples reported by Su et al. (2020), there is evidence that the SARS-CoV-2 virus containing ORF8 mutations is able to replicate and cause disease [19]. We hypothesize that the truncated ORF8 is not functional, but the overall virus fitness is not significantly affected.

### Nonstructural proteins

During SARS-CoV-2 infection, polyprotein ORF1ab is proteolytically cleaved into sixteen NSPs (NSP1-16) that collectively form the viral replication-transcriptional complex (RTC) [23]*.* ORF1a comprising NSP1-10 plays an important role in coping with cellular stresses and maintaining the functional integrity of the cellular components along with the pivotal roles in viral replication. ORF1b encodes viral RNA-dependent RNA polymerase (NSP12), helicase (NSP13), exonuclease (NSP14), a poly-U-specific endonuclease (NSP15), and methyltransferase (NSP16) [24].

NSP4 is the largest membrane protein of the NSPs, consisting of four TM spanning regions, a large luminal loop located between the first N-terminal and second TM, a second smaller luminal loop located between the third and fourth TMs, and a C-terminal cytoplasmic exposed domain. NSP4 is essential for RTC formation [25–27], and in coordination with NSP3 and NSP6 anchor the RTC to modified ER membranes [28]*.* The large luminal loop formed between the first and second transmembrane regions is a crucial component of NSP4 for ER membrane rearrangement induced by its interaction with NSP3, and disruption of this region impairs viral replication [25–27]*.* The C-terminal domain is predominately alpha-helical in structure and involved in protein–protein interactions [29]*.* The V167L and T492I NSP4 mutations located in the large luminal loop and C-terminal end, respectively, are characteristic of the Delta variant and were identified in all CO isolates. Additionally, a single CO isolate co-harbors a T60I substitution also located in the large luminal loop. While these mutations do not occur within the specific luminal loop regions involved in localization with NSP3 in other Coronaviruses [25, 26] or glycosylation sites involved in membrane structure formation [27]*,* the addition of branch-chain hydrophobic residues to the luminal loop and C-terminal end could impact protein–protein or protein-membrane interactions, suggesting these replacements likely have a potentially beneficial influence (Additional file 5). Additionally, two CO isolates are differentiated by NSP4 mutations including a premature stop-codon at position Y191 and a frameshift deletion (9124-9125nt) thereby potentially truncating the large luminal loop, last three membrane regions, and C-terminal cytoplasmic domain. While the exact functional implication of these disruptions is unknown, deletion of the large luminal loop and last three TMs has been shown to prevent localization with NSP3 [26]*.*

NSP13 is a highly conserved multifunctional protein possessing both NTPase and RNA helicase activities [23, 24]. It is comprised of a five-domain, triangular pyramid structure. The N-terminal zinc-binding domain contains three zinc-finger motifs and a bridging stalk, responsible for NSP12 interaction. The 1B domains and C-terminal RecA-like helicase domains, RecA1 and RecA2, provide the NTP and nucleic acid binding activities [24]*.* Characteristic of the Delta variant and conserved among all the CO isolates sequenced in this study is a P77L mutation located within the N-terminal zinc-binding domain required for catalytic activity and interaction with NSP12 [30]. Additionally, three CO isolates co-harbor a V226L mutation located in the 1B domain involved in substrate binding [31].

NSP14 is a bifunctional replicase consisting of both an N-terminal exoribonuclease (ExoN) and C-terminal (guanine-N7) methyl transferase (N7-MTase) domains [32]. ExoN is important for proofreading and N7-MTase functions in mRNA capping [33]. Conserved among our CO isolates and canonical to the Delta variant, is a A394V mutation located in the N7-MTase domain. Additionally, a single CO isolate co-harbors a novel non-codon-aligned insertion in NSP14 (19,486-GGT-19487) resulting in lysine and serine residues replacing valine at position 483. This mutation occurs in the N7-MTase domain and adjacent to Cys484, a residue part of the zinc-finger 3 motif [34]. This mutation could impact protein–protein interactions, but the overall effect is unknown given its’ distant location from the catalytic site and mutations in two of the motif residues only marginally affected MTase activity in other studies [34].

Two additional isolates belonging to the S:S112L sub-lineage, but outgrouping from the breakthrough cluster contain substitutions in the NSP14 ExoN domain. Both isolates were obtained and sequenced during the same April-June 2021 time period. However, one near neighbor isolate was obtained from a vaccinated individual collected in the same CO location, and the second more distantly related isolate was obtained from an individual with unknown vaccination status in a different CO location. The isolate obtained from a vaccinated individual contains tandem asparagine and arginine substitutions for isoleucine and glycine at NSP14 positions 201 and 202 respectively. While the functional implication of these polar replacements are unknown, they could impact NSP10/NSP14-ExoN complex stability [32]. The remaining isolate collected in a different CO location contains a conservative A220V mutation.

Last, NSP15 is a uridine-specific endoribonuclease composed of an N-terminal domain, a middle domain and a C-terminal endonuclease domain [35, 36]*.* NSP15 is involved with the degradation of viral RNA to evade the host defense system [35]. Located within the poly-U-specific endonuclease domain (EndoU) of NSP15, V303F introduces a potential conformational change to one of five α-helices which flank the two antiparallel β-sheets comprising the catalytic domain of NSP15, a region widely conserved among the *Coronaviridae* [35, 36]*.* While the impact of this substitution on NSP15 structure and function is unknown, the introduction of an additional aromatic residue replacing the highly conserved valine at position 303 may disorder the nearby beta-sheet structure (Additional file 6) and suggests a potentially beneficial influence (Additional file 5).

## Conclusions

There is an emerging sub-lineage of Delta subclade I isolates circulating in the U.S. Recent isolates collected from vaccinated individuals in CO have acquired distinguishing mutations when compared to other SARS-CoV-2 isolates from the same sub-lineage. Similar isolates were also identified in KS, FL, and NM. Some of these isolates may be evolving under positive selection due to their potential fitness benefit (e.g. S:S112L, NSP15:V303F and ORF7a:Q94*) resulting in their persistence and subsequent emergence of novel lineages. Whereas other less beneficial variants may be eliminated by purifying selection (e.g. ORF8:Q18*). Determining the extent to which this sub-lineage may persist and circulate more broadly could be confounded by sampling bias, lack of sampling, and lag in data reporting. As on-going genomic surveillance continues, we expect newly designated AY lineages of the Delta B.1.617.2 variant to be released, such as AY.4-AY.12 as of August 8, 2021 (PANGO v3.1.11). Additionally, experimental studies need to be conducted to confirm our hypothetical inferences.

This study is limited by its small sample size and lack of clinical data, such as vaccination status, supporting breakthrough infection samples. Therefore, associations between vaccination status and temporal relationships in the infection could not be made. The samples sequenced in this study were collected from late April to early June, when COVID-19 case numbers had declined across the U.S. and the Delta variant was emerging. The isolates collected in VA were collected within days of the first reported Delta variant in the U.S. Additionally, 28 of the 34 samples were collected from a single location in CO. This cluster of CO isolates highlights the transmissibility of the Delta variant and the importance of ongoing surveillance sequencing to public health.

## Methods

### Clinical isolates description

Thirty-four clinical nasal samples included in this study were collected in CO, VA and IA for routine SARS-CoV-2 diagnostic purposes between April 29, 2021 and June 21, 2021. The samples were obtained from a CLIA certified laboratory and confirmed to be polymerase chain reaction (PCR) positive for COVID-19. All samples with a N gene and an E gene Ct value < 36 were sequenced. Twenty-eight of the thirty-four samples were collected from vaccinated individuals.

### SARS-CoV-2 whole-genome sequencing

The viral RNA was purified from 140 µL of the clinical sample using the QIAamp Viral RNA Mini Kit (Qiagen, Hilden, Germany) following the manufacturer protocol. The cDNA was generated using random primer mix (New England BioLabs (NEB), Ipswich, MA, USA) and Superscript IV First Strand Synthesis kit (Life Technologies, Carlsbad, CA, USA). Two multiplex PCR reactions, containing a total of 17 primer pairs, previously described in Resende et al. (2021), were used to amplify across the SARS-CoV-2 genome. Primer hCov_F1 was replaced with hCov_F1Alt1 to achieve additional coverage compared to the previously described primer design (Additional file 7) [37]. Each primer pair produces an amplicon approximately 1,900 base pairs (bp) in size with an average of 175 bp overlap between the amplicons. The PCR was performed using the Q5 High-Fidelity DNA Polymerase (NEB, Ipswich, MA, USA). The amplicons from both primer pools were combined. The pooled amplicon was purified using Agencourt AMPure XP beads (Beckman Coulter, Brea, CA, USA) and quantified using the Qubit 4.0 Fluorometer and the Qubit double-stranded High Sensitivity kit (Life Technologies, Carlsbad, CA, USA). The purified PCR amplicon was diluted to 4.8 ng/µL for library preparation. A total of 60 ng of PCR amplicon was treated with Ultra II End Prep Enzyme mix (NEB, Ipswich, MA, USA). After end repair, the Native Barcodes 1–24 (Oxford Nanopore Technologies (ONT), Oxford, UK) were ligated using the Ultra II Ligation Module (NEB, Ipswich, MA, USA). Library preparation for sequencing on the ONT MinION using the Ligation Sequencing 1D kit, SQK-LSK-109 (ONT, Oxford, UK). A total of 24 samples, including a positive and negative control, were pooled for sequencing on the same flow cell. Sequencing was performed on the MinION using the R9.4.1 flow cell (ONT, Oxford, UK) for 16 to 20 h.

### SARS-CoV-2 genome analysis

Raw nanopore signal was processed using ONT’s Guppy basecaller in high-accuracy mode using a single Nvidia Tesla V100 GPU. The basecalled reads were demultiplexed using ONT’s Guppy barcoder to bioinformatically separate the reads into their appropriate samples. Unclassified reads were discarded. Reads were filtered to a minimum size of 1,500 base pairs (bp) and a maximum size of 3,500 bp using artic guppyplex. This process was executed according to the artic protocol (https://artic.network/ncov-2019/ncov2019-bioinformatics-sop.html). Reads were aligned to the SARS-CoV-2 Wuhan-Hu-1 reference genome (GenBank sequence MN908947.3) using MinMap2 via ONT’s medaka consensus pipeline. Variants were annotated using SnpEff v5.0 [38] and functional impacts were predicted with SNAP2 [39]*.* Mutations were validated using ONT’s medaka tools and annotated with a filtering threshold of DPSP + -25 bp > 20x. Mutations with a DPSP > 20 × and Phred base score < 8.0 were manually reconciled (Additional file 8). Pangolin COVID-19 Lineage Assigner [40] was used to assign B.1.617.2 SARS-CoV-2 phylogenetic lineages [41]*.* Complete, high-coverage SARS-CoV-2 genomes classified as belonging to the B.1.617.2 lineage (including AY.1, AY.2, and AY.3) and obtained in the U.S. were downloaded from GISIAD (https://www.gisaid.org/) on July 6, 2021 (*n* = 4,479). Genomes were trimmed relative to positions 102 and 29,740 in the reference genome. All genomes containing < 1% Ns and a final trimmed length > 29560 bp were included in downstream analyses. All genomes were confirmed as PANGO-assigned Delta variants. The resulting 1,318 unique high-quality genomes were aligned to the reference genome using MAFFT v7.471 using the FFT-NS-2 iterative refinement method [42]*.* Multiple sequence alignments were rendered using ESPript 3.0 [43]*.* A maximum-likelihood phylogenetic tree using IQ-TREE v2.0.3 [44] was estimated using the GTR + F + I model of nucleotide substitution and ultrafast bootstrapping with 1000 replicates. The resulting tree was rendered with iTOL Interactive Tree of Life [45]*.* To identify a representative subset of S:S112L sub-lineage genomes for comparison, downloaded GISAID genomes belonging to this sub-lineage were compared using a fast Average Nucleotide Identity (ANI) estimate generated using MASH [46] and GGRaSP [47] to choose a single medoid sequence from any complete linkage ANI cluster with a threshold of 0.04% or 4/10,000 base pair difference. The resulting genomes were then aligned to the reference genome using MAFFT [42] and trimmed relative to positions 102 and 29,740 in the reference genome, and a phylogenetic tree using IQ-TREE v2.0.3 [44] was estimated and rendered with iTOL Interactive Tree of Life [45] using the methods described previously. Molecular graphics and analyses of protein structures were performed with UCSF ChimeraX [48].

## Supplementary Information

**Additional file 1.** Sequence and assembly summary and metadata for all SARS-CoV-2 genomes sequenced as part of this study.

**Additional file 2.** Above: Amino acid sequence alignment of S protein from SARS-CoV-2 (GenBank sequence MN908947.3) and representatives isolates sequenced in this study labeled by collection locaton. Secondary structure elements from PDB 6VXX displayed above at positions 14-1211 and relative accessibility (acc) of each residue and hydrophobicity (hyd) are displayed below. Mutations discussed in this study are highlighted in yellow. Below: Shown is the ribbon structure of a single monomer of SARS-CoV-2 Spike protein, labeling amino acid changes in common on Delta variant lineaged in a lime color (Thr95Ile, Gly142Asp, Leu452Arg, Asp614Gly, Pro681Arg, Asp950Asn). The mutation at position Ser112Leu is shown labeled red. The Spike protein structure shown was obtained from PDB:6VXX. This structure does contain structural information for three other variant amino acid positions 19, 156-158.

**Additional file 3.** Amino acid sequence alignment of ORF7a from SARS-CoV-2 (GenBank squence MN908947.3), SARS-CoV (GenBank sequence NC_004718.3), and representative isolates sequenced in this study labeled by collection location. Secondary structure elements from PDB 6W37 displayed above at positions 15-81 and relative accessibility (acc) of each residue and hydrophobicity (hyd) are displayed below. Mutations discussed in this study are highlighted in yellow.

**Additional file 4.** Above: Amino acid sequence alignment of ORF8 from SARS-CoV-2 (GenBank sequence MN908947.9) and representative isolates sequenced in this study labeled by collection location. Secondary structure elements fron PDB 7JTL displayed above at positions 19-121 and relative accessibility (acc) of each residue and hydrophobicity (hyd) are displayed. Mutations discussed in this study are highlighted in yellow. Bellow: Shown is the ribbon structure of a SARS-CoV-2 accessory protien ORF8, labeling amino acid deletion observed in 35 variants in lime color. Five of the strains analyzed have a premature stop mutation G1n18* leading to the loss of the ORF8 polypeptide beyond the prdicted signal peptide sequence (represented with the red ribbon backone color). The ORF8 structure shown was obtained from PDB:7TJL.

**Additional file 5.** Functional effects of mutations identified in SARS-CoV-2 genomes sequenced in this study.

**Additional file 6.** Above: Amino acid sequence alignment of endoU domain from NSP15 homologs from representative members of the *Coronoviridae* sub-family and isolates sequenced in this study labelled by collection location. Secondary structure elements from PDB 6WLC displayed above and relative accessibility (acc) of each residue and hydrophobicity (hyd) are displayed below the alignment. Mutations discussed in this study are highligted in yellow. Below: The ribbon structured of NSP15 endoribonuclease from SARS-CoV-2 PDB 6WLC with the endoU domain indicated in the sequence alignment above shown in red. Within the red region a single green amino acid is highlighted at V303F.

**Additional file 7.** Primers utilized in amplification.

**Additional file 8.** Variants observed in SARS-CoV-2 genomes sequenced in this study.

**Additional file 9.** GISAID genomes sequences used in this study.

## Data Availability

The whole genomes sequenced and analyzed in this study, as well as their associated metadata, are available at NCBI GenBank under BioProject no. PRJNA718231 with the following BioSample no. SAMN20863165 to SAMN20863198 as well as deposited at GISAID (Additional file 1). GISAID publicly available sequences used for comparison are in Additional file 9.

## Abbreviations

ACE2: Angiotensin Converting Enzyme 2
ANI: Average Nucleotide Identity
CLIA: Clinical Laboratory Improvement Amendments
COVID-19: Coronavirus Disease 2019
Ct: Cycle Threshold (Ct)
DPSP: Depth of Reads Spanning Position
EndoU: Endonuclease
ExoN: Exoribonuclease
GISAID: Global Initiative on Sharing Avian Influenza Data
HIPAA: Health Insurance Portability and Accountability Act of 1996
IRB: Institutional Review Board
N7-MTase: (Guanine-N7) Methyl Transferase
NSP: Nonstructural Protein
ONT: Oxford Nanopore Technologies
ORF: Open Reading Frame
PCR: Polymerase Chain Reaction
RDB: Receptor Binding Domain
RdRp: RNA-dependent RNA polymerase
SARS-CoV-2: Severe Acute Respiratory Syndrome Coronavirus 2
