# Supplementary figures and images for: SARS-CoV-2 Delta variant isolates from vaccinated individuals
Source: BMC Genomics. 2022 Jun 4;23:417. doi: 10.1186/s12864-022-08652-z (PMC9166184; doi:10.1186/s12864-022-08652-z)

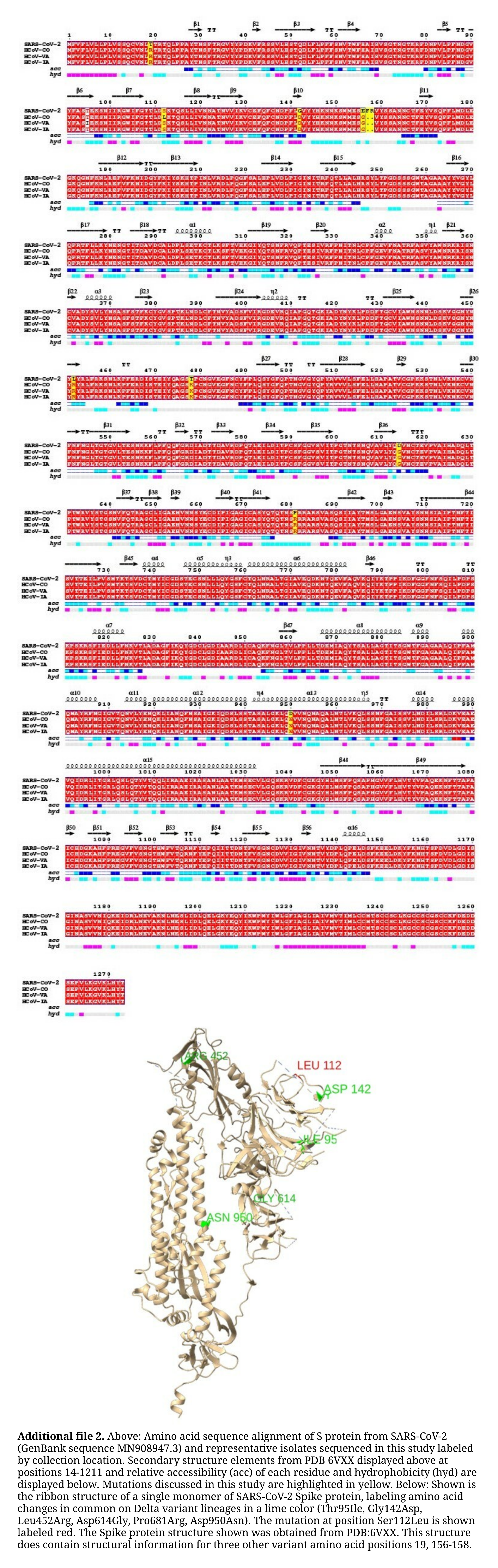

Supplement: Supplementary file 2 — Additional file 2. Above: Amino acid sequence alignment of S protein from SARS-CoV-2 (GenBank sequence MN908947.3) and representatives isolates sequenced in this study labeled by collection locaton. Secondary structure elements from PDB 6VXX displayed above at positions 14-1211 and relative accessibility (acc) of each residue and hydrophobicity (hyd) are displayed below. Mutations discussed in this study are highlighted in yellow. Below: Shown is the ribbon structure of a single monomer of SARS-CoV-2 Spike protein, labeling amino acid changes in common on Delta variant lineaged in a lime color (Thr95Ile, Gly142Asp, Leu452Arg, Asp614Gly, Pro681Arg, Asp950Asn). The mutation at position Ser112Leu is shown labeled red. The Spike protein structure shown was obtained from PDB:6VXX. This structure does contain structural information for three other variant amino acid positions 19, 156-158. [file 12864_2022_8652_MOESM2_ESM.png]

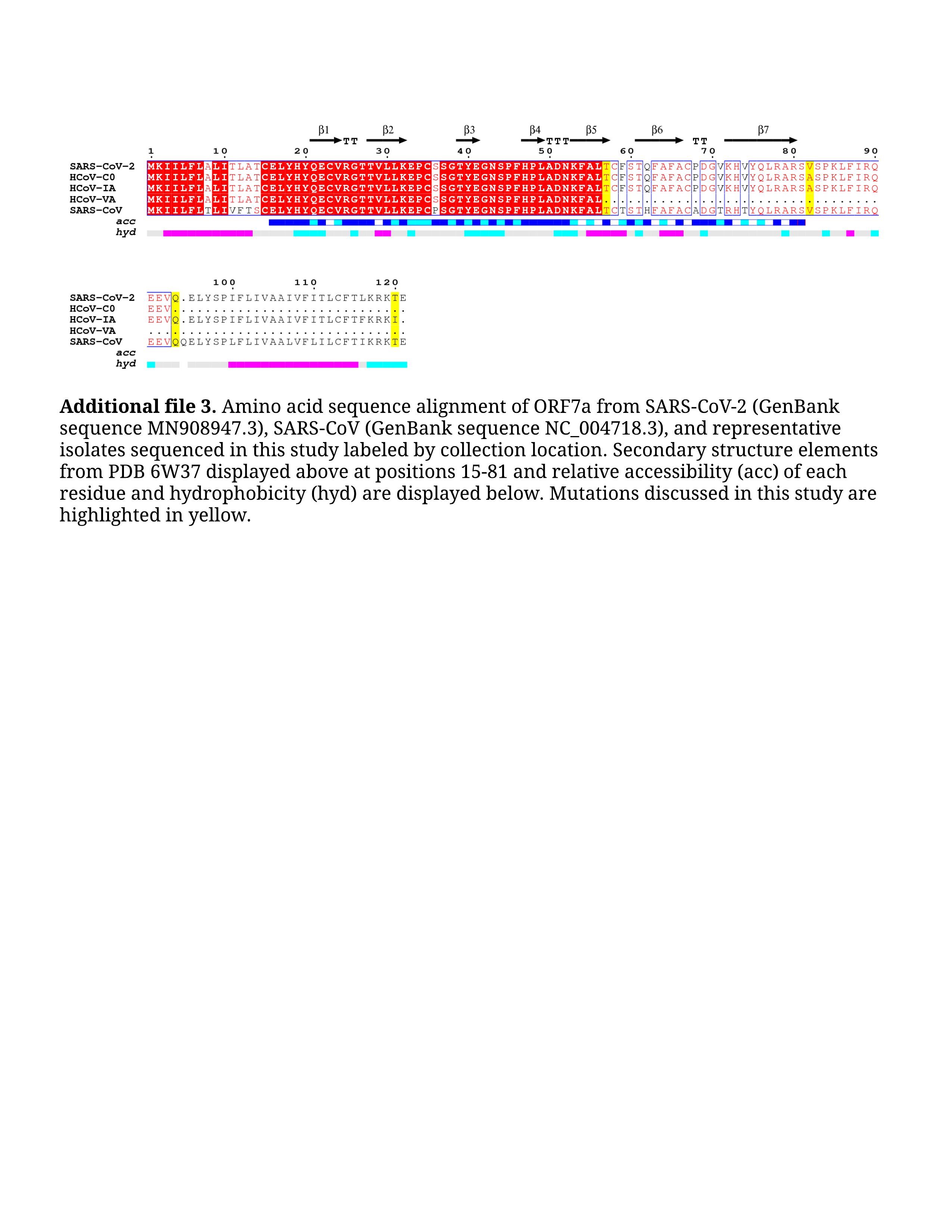

Supplement: Supplementary file 3 — Additional file 3. Amino acid sequence alignment of ORF7a from SARS-CoV-2 (GenBank squence MN908947.3), SARS-CoV (GenBank sequence NC_004718.3), and representative isolates sequenced in this study labeled by collection location. Secondary structure elements from PDB 6W37 displayed above at positions 15-81 and relative accessibility (acc) of each residue and hydrophobicity (hyd) are displayed below. Mutations discussed in this study are highlighted in yellow. [file 12864_2022_8652_MOESM3_ESM.png]

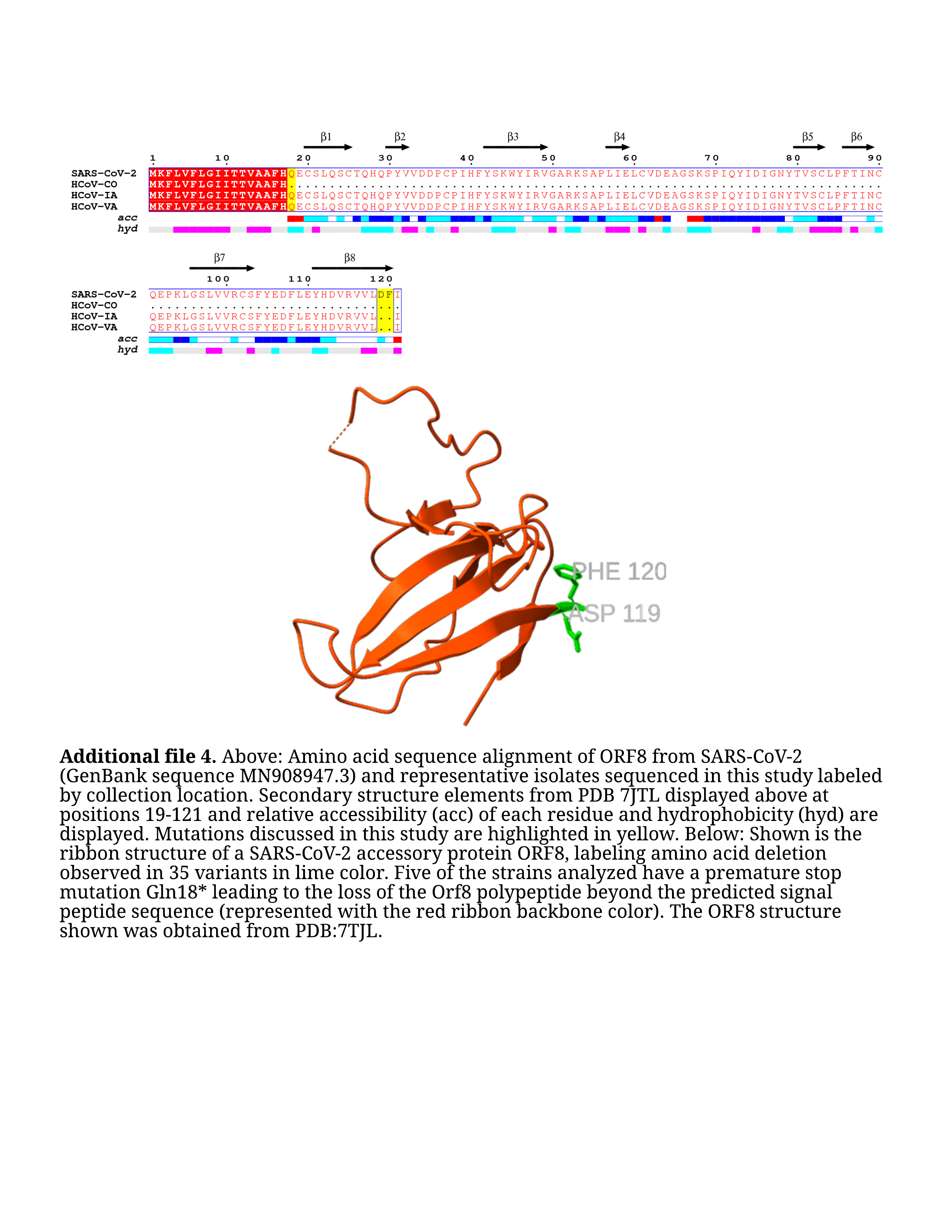

Supplement: Supplementary file 4 — Additional file 4. Above: Amino acid sequence alignment of ORF8 from SARS-CoV-2 (GenBank sequence MN908947.9) and representative isolates sequenced in this study labeled by collection location. Secondary structure elements fron PDB 7JTL displayed above at positions 19-121 and relative accessibility (acc) of each residue and hydrophobicity (hyd) are displayed. Mutations discussed in this study are highlighted in yellow. Bellow: Shown is the ribbon structure of a SARS-CoV-2 accessory protien ORF8, labeling amino acid deletion observed in 35 variants in lime color. Five of the strains analyzed have a premature stop mutation G1n18* leading to the loss of the ORF8 polypeptide beyond the prdicted signal peptide sequence (represented with the red ribbon backone color). The ORF8 structure shown was obtained from PDB:7TJL. [file 12864_2022_8652_MOESM4_ESM.png]

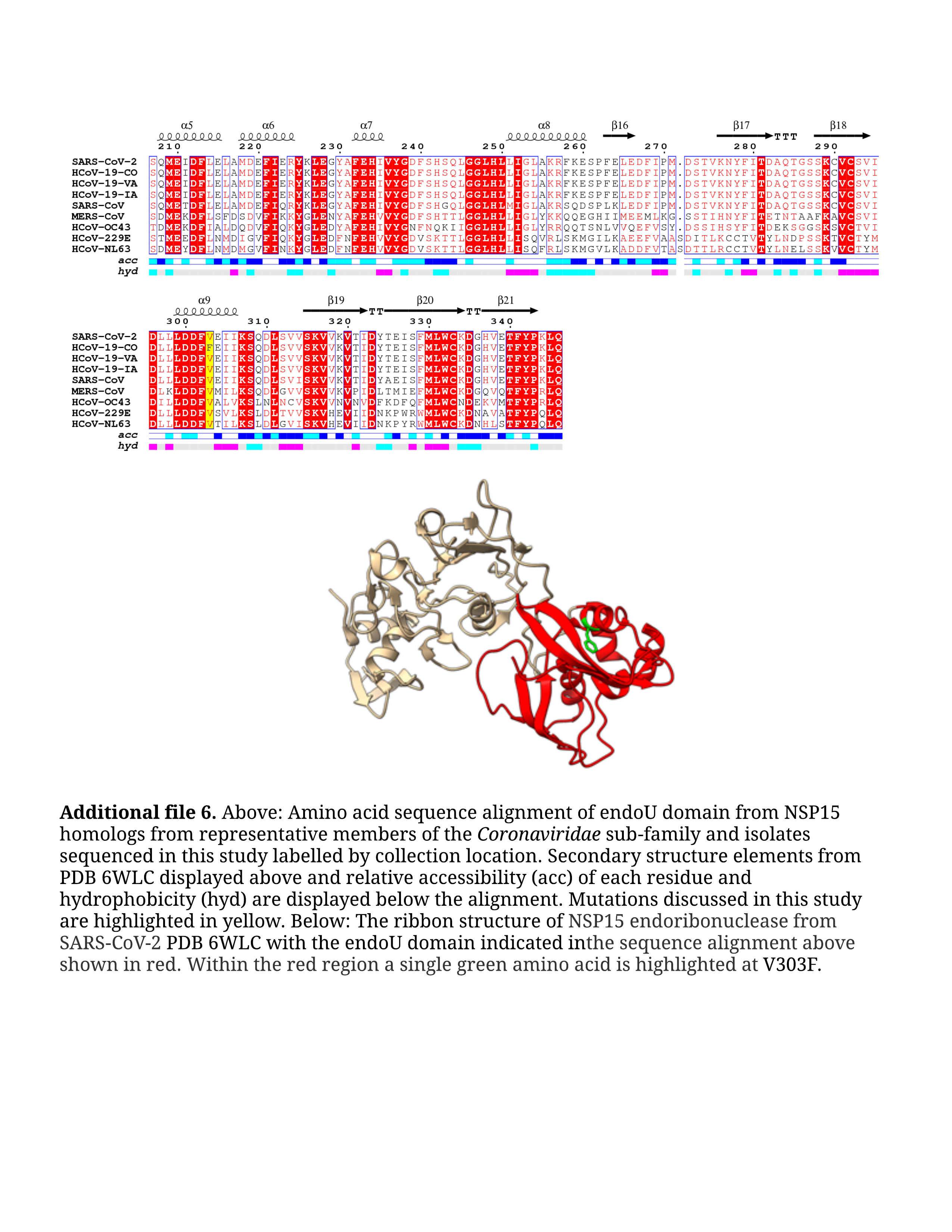

Supplement: Supplementary file 6 — Additional file 6. Above: Amino acid sequence alignment of endoU domain from NSP15 homologs from representative members of the Coronoviridae sub-family and isolates sequenced in this study labelled by collection location. Secondary structure elements from PDB 6WLC displayed above and relative accessibility (acc) of each residue and hydrophobicity (hyd) are displayed below the alignment. Mutations discussed in this study are highligted in yellow. Below: The ribbon structured of NSP15 endoribonuclease from SARS-CoV-2 PDB 6WLC with the endoU domain indicated in the sequence alignment above shown in red. Within the red region a single green amino acid is highlighted at V303F. [file 12864_2022_8652_MOESM6_ESM.png]
